# Correction: Morzhin, O.V.; Pechen, A.N. Control of the von Neumann Entropy for an Open Two-Qubit System Using Coherent and Incoherent Drives. *Entropy* 2024, *26*, 36

**DOI:** 10.3390/e26090750

**Published:** 2024-09-02

**Authors:** Oleg V. Morzhin, Alexander N. Pechen

**Affiliations:** Department of Mathematical Methods for Quantum Technologies & Steklov International Mathematical Center, Steklov Mathematical Institute of Russian Academy of Sciences, 8 Gubkina Str., 119991 Moscow, Russia

In the published article [1], the authors need to remove the second affiliation. Also, there was an error regarding the first affiliation. The updated affiliation should be: Department of Mathematical Methods for Quantum Technologies & Steklov International Mathematical Center, Steklov Mathematical Institute of Russian Academy of Sciences, 8 Gubkina Str., 119991 Moscow, Russia. The authors state that the scientific conclusions are unaffected. This correction was approved by the Academic Editor. The original publication has also been updated.
